# Extensive subcutaneous emphysema and intra-abdominal sepsis following haemorrhoid operation: a rare complication of a novel procedure

**DOI:** 10.1093/jscr/rjad177

**Published:** 2023-04-12

**Authors:** Jennifer D Turco, Anand Trivedi

**Affiliations:** Fiona Stanley Hospital, Department of General Surgery, Murdoch WA, Australia; Fiona Stanley Hospital, Department of General Surgery, Murdoch WA, Australia

**Keywords:** HAL-RAR, haemorrhoidal artery ligation, rectal perforation, haemorrhoids

## Abstract

Doppler-guided haemorrhoidal artery ligation and recto anal repair (HAL-RAR) procedure is a relatively new, minimally invasive procedure for the treatment of Grades III and IV haemorrhoids. A 71-year-old female presented with sepsis, abdominal distension and extensive subcutaneous emphysema and was found to have intra- and extraperitoneal rectal perforation requiring repair, laparoscopy and sigmoid colostomy. Suture ligation of the haemorrhoidal artery can inadvertently be above the peritoneal reflection and result in full thickness rectal perforation secondary to ischaemic necrosis. Previous vaginal prolapse mesh repair should be considered as a relative contraindication to HAL-RAR as it can significantly distort the anatomy.

## INTRODUCTION

The haemorrhoidal artery ligation and recto anal repair (HAL-RAR) is a relatively new, minimally invasive technique for the treatment of Grades III and IV haemorrhoids, using doppler-guided haemorrhoidal artery ligation (HAL) combined with the mucopexy of the prolapsed haemorrhoidal tissue (recto anal repair). HAL was first described by Morinaga *et al*. in 1995 [1]. Since then, many similar devices have been developed for HAL combined with mucopexy if required. The HAL-RAR procedure uses a special proctoscope equipped with a doppler transducer to localise and ligate the submucosal distal branches of the superior rectal artery with a ‘figure of eight’ stitch placed under vision at a depth of 3 and 6 mm from the surface, 3–4 cm above the dentate line [2]. After each ligation, the proctoscope can be rotated to localise other branches. Mucopexy is then performed selectively on prolapsing haemorrhoids, using the same proctoscope, with continuous running sutures applied longitudinally proximal to distal in the lower part of the rectum. Tying the suture lifts the prolapsed mucosa back into the rectum. No tissue is excised. It is considered safe and effective technique with high success and low complication and recurrence rates [3, 4].

## CASE REPORT

A 71-year-old female underwent an HAL-RAR procedure for Grade III haemorrhoids at a peripheral hospital. Her comorbidities included hypertension, chronic back pain and gasrto-oesophageal reflux disease (GORD). Her past surgical history included a vaginal prolapse repaired with mesh. She was a non-smoker and independent with her activities of daily living. On post-operative Day 2, she felt unwell and complained of swelling in her neck and crackling sensation in her chest, neck and cheeks.

On physical examination, the patient was speaking in full sentences and oxygen saturations were 97% on room air. She was tachycardic (120 bpm), normotensive (150/90 mmHg) and afebrile. She was found to have extensive subcutaneous emphysema over her chest, neck and maxilla. Her abdomen was mildly distended with no peritonism. A digital rectal exam was not performed because of concern for perforation.

### Diagnostic assessment

**Figure 1:**
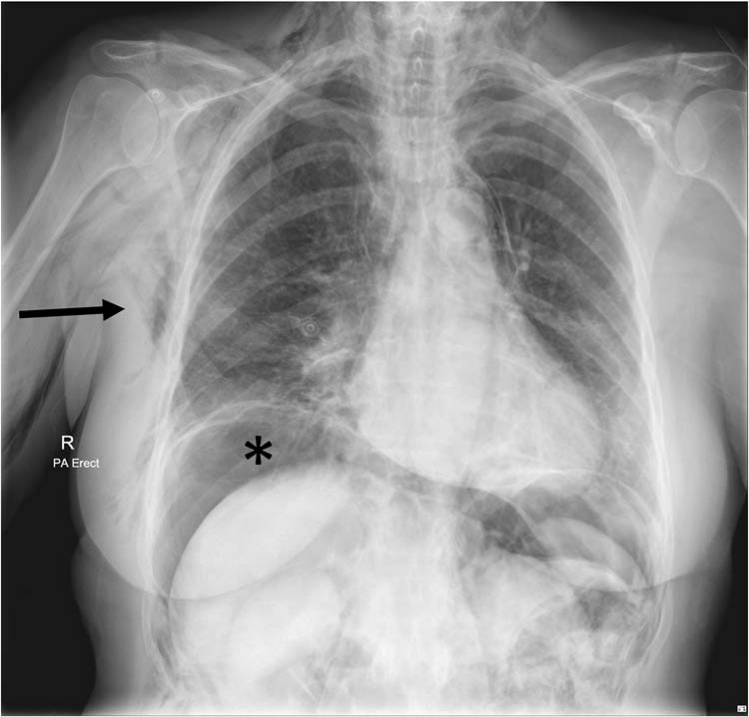
Chest X-ray Day 2 post-operatively, showing extensive subcutaneous emphysema on the right (arrow) and subdiaphragmatic free gas (*) concerning for perforated viscus.

**Figure 2:**
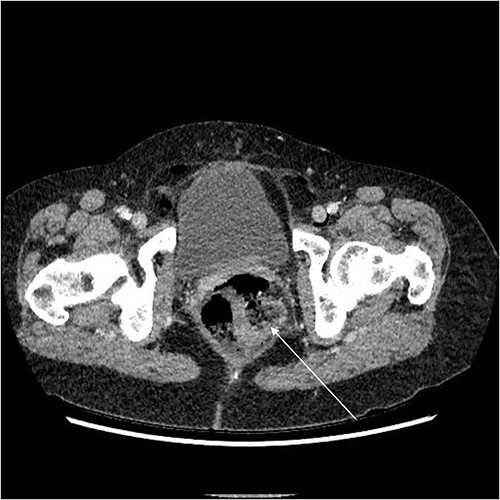
Axial CT showing rectal perforation (arrow).

**Figure 3:**
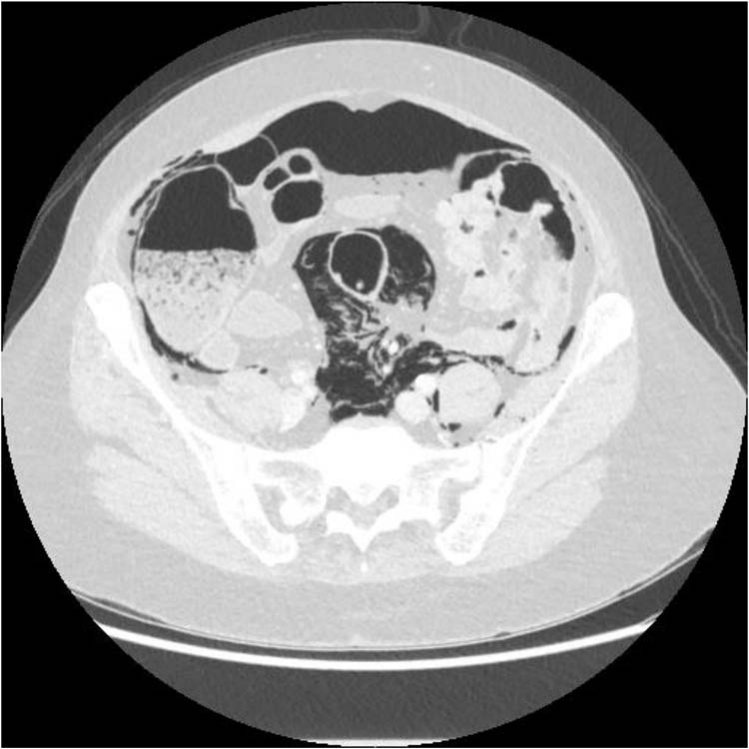
Axial CT showing extensive intra- and extraperitoneal free gas.

A chest X-ray confirmed subcutaneous emphysema and pneumoperitoneum and no pneumothorax (Fig. 1). Subsequent computed tomography (CT) of the abdomen and pelvis revealed extensive extraluminal gas throughout neck, torso and upper limbs including retroperitoneal, intraperitoneal, mediastinum, anterior and posterior chest wall, superficial and deep layers of the neck, right lower abdominal wall and bilateral inguinal canals (Figs 2 and 3).

### Therapeutic intervention

**Figure 4:**
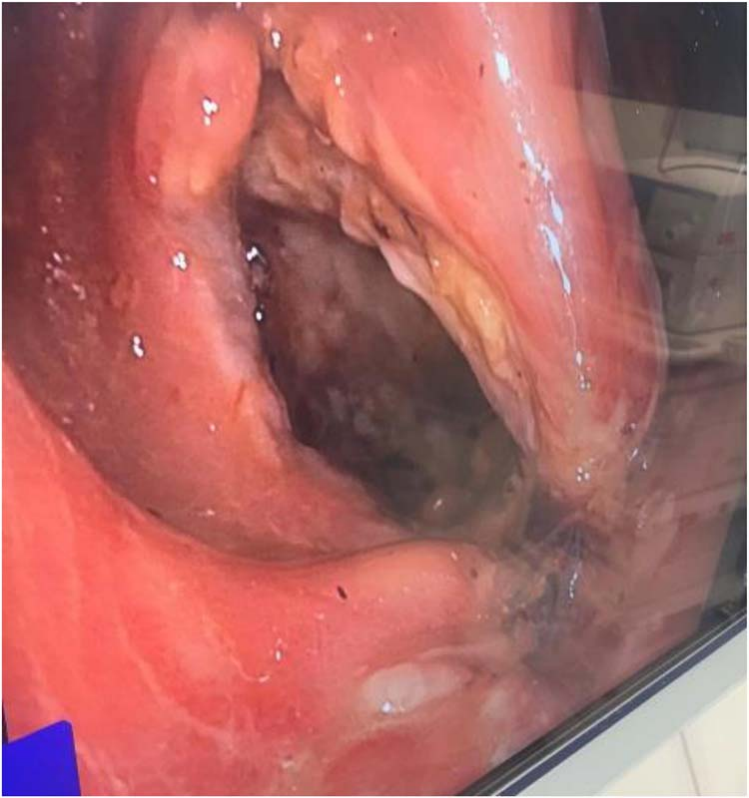
Flexible sigmoidoscopy image of full thickness rectal perforation.

The patient was immediately transferred from a peripheral hospital to a tertiary hospital where she was assessed by the surgical team and promptly taken to theatre for an examination under anaesthetic and flexible sigmoidoscopy, which revealed a large full thickness defect in the rectum, 3–4 cm from the anal verge, anterolaterally to the right, between the sutures for hemorrhoidal ligation (Fig. 4). The rectal perforation lead into a large extraperitoneal cavity that was washed out and a Foley catheter left in for ongoing drainage.

Laparoscopy revealed extensive free gas in peritoneal cavity on entry and extensive retroperitoneal gas on right side and surrounding rectum. There was no peritoneal fluid or contamination seen. An Abcarian sigmoid colostomy was formed.

### Follow-up and outcomes

The patient recovered well post-operatively, after initial damage control measures. Six weeks later, she underwent the removal of the remaining upper posterior vaginal wall mesh, closure of rectal perforation and a posterior vaginal repair for a large rectocele by a consultant urogynaecologist. Four months post the original damage control procedure, the patient underwent closure of the sigmoid colostomy and repair of parastomal hernia by consultant colorectal surgeon.

## DISCUSSION

HAL-RAR is indicated in Grades III and IV haemorrhoids. There are no well-documented contraindications.

Recent studies have reported a high success rate with no pain reported at 1 month, complete resolution of symptoms in 85% of patients at 1 month and 92% at 12 months and a complication rate of only 7.6%. Complications reported include urinary retention (2%), dyschezia (3%), bleeding (2%) and haemorrhoidal necrosis (1%) [5]. A prospective analysis of 30 consecutive cases published in 2016 has 93% success rate and only one complication (recurrence) at 24 month follow-up. Although urinary retention, bleeding, tenesmus, anal fissure and infection are known complications, there are no cases of rectal perforation as a complication of HAL-RAR in the limited published literature.

Though this novel procedure for management of haemorrhoids has been described in literature, with generous success rates and less complications, we believe that complications like these can be highly under-reported. To our knowledge, there are no reported cases of rectal perforation with combined extra and intra peritoneal breach following an HAL-RAR procedure.

## CONCLUSIONS

Haemorhoid artery ligation can occur above the peritoneal reflection using this newer technique, resulting in necrosis and perforation. We propose that HAL-RAR should not be performed in patients that may have distorted anatomy such as previous vaginal prolapse repair with mesh or rectocele. We advocate that meticulous care should be exercised while ligating haemorrhoidal artery as ischemic necrosis can lead to disastrous complications from a simple procedure. Any patients who deviate from normal post-operative course should have a high index of suspicion for rectal wall injury.
